# Tff3, as a Novel Peptide, Regulates Hepatic Glucose Metabolism

**DOI:** 10.1371/journal.pone.0075240

**Published:** 2013-09-23

**Authors:** Yuan Xue, Lian Shen, Ying Cui, Huabing Zhang, Qi Chen, Anfang Cui, Fude Fang, Yongsheng Chang

**Affiliations:** National Laboratory of Medical Molecular Biology, Institute of Basic Medical Sciences, Chinese Academy of Medical Sciences and Peking Union Medical College, Beijing, China; Broad Institute of Harvard and MIT, United States of America

## Abstract

Type 2 diabetes mellitus (T2DM) is a chronic metabolic disorder strongly associated with hepatic glucose intolerance and insulin resistance. The trefoil peptides are a family of small regulatory proteins and Tff3 is widely expressed in multiple tissues including liver. But the roles of Tff3 in regulation of glucose metabolism and insulin sensitivity in liver remain unclear. Here we show that the hepatic Tff3 expression levels were decreased in ob/ob and high-fat diet-induced obese mice. Overexpression of Tff3 in primary mouse hepatocytes inhibited the expression of gluconeogenic genes, including G6pc, PEPCK and PGC-1α, subsequently decreasing cellular glucose output. GTT and ITT experiments revealed that adenovirus-mediated overexpression of Tff3 in diabetic or obese mice improved glucose tolerance and insulin sensitivity. Collectively, our results indicated that Tff3 peptides are involved in glucose homeostasis and insulin sensitivity, providing a promising peptide on new therapies against the metabolic disorders associated with T2DM.

## Introduction

Type 2 diabetes mellitus (T2DM) is a chronic metabolic disorder strongly associated with hepatic glucose intolerance and insulin resistance. Diabetes affected estimated 366 million people in world in 2011 and the number of diabetic patients will rise to 552 million by 2030 [1]. It is serious that diabetes is becoming a major cause for morbidity and mortality, leading to a commensurate increase in the social and economic costs [2]. Maintenance of plasma glucose homeostasis is vital for survival of mammalian organisms and hepatic gluconeogenesis is absolutely required for maintaining glucose homeostasis during prolonged fasting. Peroxisome proliferator-activated receptorγ coactivator-1α (PGC-1α) is a multifunctional transcriptional coactivator involved in many metabolic pathways. Under fasting condition PGC-1α expression in liver is induced to stimulate the expression of gluconeogenic genes through directly interacting with and coactivating hepatic transcription factors such as HNF4α, PPARα, GR and FOXO1 [3-5]. Adenovirus-mediated overexpression of PGC-1αin primary hepatocytes is sufficient to drive the expression of gluconeogenic genes, such as G6pc (glucose-6-phosphatase) and PEPCK (phosphoenolpyruvate carboxykinase), encoding the key enzymes in the synthesis of glucose in livers that are regulated by a variety of dietary and hormonal signals [6,7]. However, the abnormal activation of hepatic gluconeogenesis contributes to elevation of glucose levels and the inhibition of hepatic gluconeogenesis provide effective strategies for treating T2DM [8,9].

In mammals the trefoil factor (TFF) family comprises 3 members: Tff1 (originally termed pS2), Tff2 (also called hSP) and Tff3 (formerly called ITF) [10]. These proteins are small and compact peptides containing one or two trefoil domains. The compact structure makes TFFs relatively stable to proteolytic degradation in the stomach and small intestine. Tff2 has two trefoil domains, while Tff1 and Tff3 contain only one trefoil domain. However, both Tff1 and Tff3 can dimerize to homodimers via a seventh cysteine residue [11]. Current evidences suggest that these trefoil peptides are involved in mucosal surface maintenance and restitution [12]. Tff3 was identified to be associated with cell proliferation and differentiation [13,14]. Further studies indicate that Tff3 promotes restoration of intestinal epithelium through inhibiting apoptosis and promoting survival and migration of epithelial cells into lesions [15,16]. Human Tff3 contains 59 amino acids and its molecular weight is 6.6kD (monomer) or 13kD (dimer) [17,18].

Tff3 as a novel secretory protein is the most widely expressed peptide of the three TFFs and produced by colon, pancreas, trachea, small intestine, spleen, liver, lung and stomach [14]. Interestingly, Tff3 regulates a pathway of β-cell replication, which can be exploited for expansion or preservation of functional islet β-cell mass [19]. Tff3 knockout mice display increased sensitivity to chemically induced mucosal injury due to impairment of mucosal healing and decreases in epithelial regeneration [20] and reduced synthesis of both gastric Tff1 and Tff2 [21]. Tff3 are transcriptionally regulated in response to hypotonicity and ethanol in gastric and intestinal cell lines [22]. Expression and secretion of Tff3 is up-regulated by neuropeptides and acetylcholine in the intestine cell line HT29, indicating that Tff3 regulation is integrated into the enteric neuroendocrine system [23]. A reduced Tff3 expression is proposed to be associated with fasting-induced gut mucosal atrophy [24].

However, the functions of Tff3 in regulation of hepatic glucose metabolism and insulin sensitivity have not yet been explored. Interestingly, our microarray analysis of gene expression in the livers of obese mice identified that Tff3 expression levels were decreased. Thus, we hypothesized that decreased Tff3 expression in livers is associated with abnormal glucose metabolism in these obese mice. We generated adenovirus expressing Tff3 and injected it into diabetic mice. As results, we found that adenovirus-mediated overexpression of Tff3 in the livers of diabetic or obese mice inhibited the expression of gluconeogenic genes, thereby decreasing blood glucose levels and improving glucose tolerance. Collectively, our results indicate that secreted peptide Tff3 is involved in hepatic glucose homeostasis, providing a promising new lead for developing therapies against the metabolic disorders associated with T2DM.

## Experimental Procedures

### 2.1 Experimental animals

Male, 8-week-old C57BL/6 mice, male Lepr ^db^/Lepr^db^ (db/db) mice and Lepr ^ob^/Lepr^ob^ (ob/ob) were purchased from the Model Animal Research Center of Nanjing University (Nanjing, China). Mice were housed and maintained on a 12-hr light-dark cycle with regular unrestricted diet, and fed with either a normal chow (9% fat; Lab Diet) or an HF diet (45% fat; Research Diets, CA) and libitum with free access to water. All animal experiments were carried out under protocols approved by the Animal Research Committee in the Institute of Laboratory Animals, Chinese Academy of Medical Sciences and Peking Union Medical College. All the surgeries were performed under sodium pentobarbital anesthesia, and all efforts were made to minimize suffering. Mice were injected with adenovirus-containing green fluorescent protein (Ad-GFP), Ad-Tff3 (0.5-1.0×10^9^ active viral particles in 200µl of PBS), and 5-7 days after infection, mice were fasted for 6h, and livers were collected for further analysis. Blood glucose was measured using a One-Touch Ultra^®^ glucometer (LifeScan Inc., Milpitas, CA). After an overnight (16h) fast, mice underwent Glucose Tolerance Tests (GTT) with intraperitoneal injection of 1g/kg glucose for DIO, db/db or ob/ob mice. Blood glucose was measured 0, 15, 30, 45, 60 and 90 min after glucose injection. In Insulin Tolerance Tests (ITT), mice were fasted for 6h and human insulin was injected at a dose of 0.75 U/kg for DIO, db/db or ob/ob mice. Blood glucose was monitored 0, 15, 30, 45, 60 and 90min after insulin injection. In pyruvate tolerance test, mice were fasted for 16h before injection with 0.5g/kg pyruvate. Blood glucose was monitored at the same time point above.

### 2.2 Construction and purification of adenoviruses expressing Tff3

Tff3 gene was ampliﬁed by polymerase chain reaction (PCR) from the liver cDNA of C57BL/6J mouse and cloned into an entry vector using the following PCR primer pairs: 5’- TCCTGAAGCTTGCCTGCTGCC-3’ (forward) and 5’- AGCAGGGAGCAGATCGGGGA-3’ (reverse). Then Tff3 coding sequence was recombined into the Gateway-based pAd-Track-CMV vector (Invitrogen) according to the manufacturer’s instructions. Adenovirus expressing GFP and Tff3 were generated as previously described [25]. Amplification of recombinant adenovirus was performed according to the manufacturer’s instructions (Invitrogen) using HEK 293A cells. Viruses were purified by the cesium chloride method and dialyzed against phosphate-buffered-saline buffer containing 10% glycerol prior to animal injection.

### 2.3 Primary hepatocytes studies

Mouse primary hepatocytes were obtained from C57BL/6 mice. Cells were plated onto 6-well, collagen-coated dishes, and viability was estimated by trypan blue exclusion at>80%. Mouse hepatocytes were cultured in RPMI-1640 containing 10% FBS, 100 units/ml penicillin, and 100g/ml streptomycin. Mouse hepatocytes were infected with the adenoviruses. Cells were harvested 1-2 days after infection as indicated. For HGP (hepatic glucose production) assays, after primary mouse hepatocytes were infected for 30h, cells were washed 3 times with PBS. Then cells were incubated in 2 ml/well of phenol red–free, glucose-free DMEM containing 1 µM dexamethasone (DEX), 2 mM pyruvate, 20 mM lactate, and 10 µM forskolin (FSK). The medium was collected 3 hours later, and 0.5ml of medium was taken to measure the glucose concentration in the culture medium using a glucose assay kit (Applygen Technologies Inc). A 2-fold concentration of the kit reagents was used to increase the sensitivity. Cells were collected and lysed, and the total protein concentration was measured (Bio-Rad) to correct cell count.

### 2.4 Real-time PCR

Total RNA was extracted with Trizol (Life Technologies Inc., Carlsbad, CA). The cDNA was synthesized using random primers and High Capacity cDNA Reverse Transcription Kit (Applied Biosystems). Quantitative real-time reverse-transcriptase PCR (qRT-PCR) was performed using Go Taq qPCR master mix (Promega) and specific primers (Table S1) on a Bio-Rad IQ5 system. The quantitative results were normalized by the levels of β-actin mRNA.

### 2.5 Western blot

Lysates or conditioned media were homogenized on ice in lysis buffer and centrifuged, and aliquots diluted with 5× electrophoresis buffer. Protein was separated on a 10% SDS-polyacrylamide gradient gel (BioRad, CA). Antibodies include Akt (1:1000), p-Akt (Ser473) (1:700), GSK3β (1:1000), p-GSK3β (1:700) from Cell Signaling Technologies; PGC-1α (1:700), GAPDH (1:5000) from Abcam; Tff3 (1:100), C-myc (1:5000) from Santa Cruz Biotechnology.

### 2.6 Statistical analysis

Data are presented as means +SEM and compared between or among groups by a two-tailed unpaired Student *t* test or by one-way ANOVA followed by a Fisher least significant difference test. *P*< 0.05 was considered statistically significant.

## Results

### Expression levels of Tff3 in livers of obese mice are decreased

**Figure 1:**
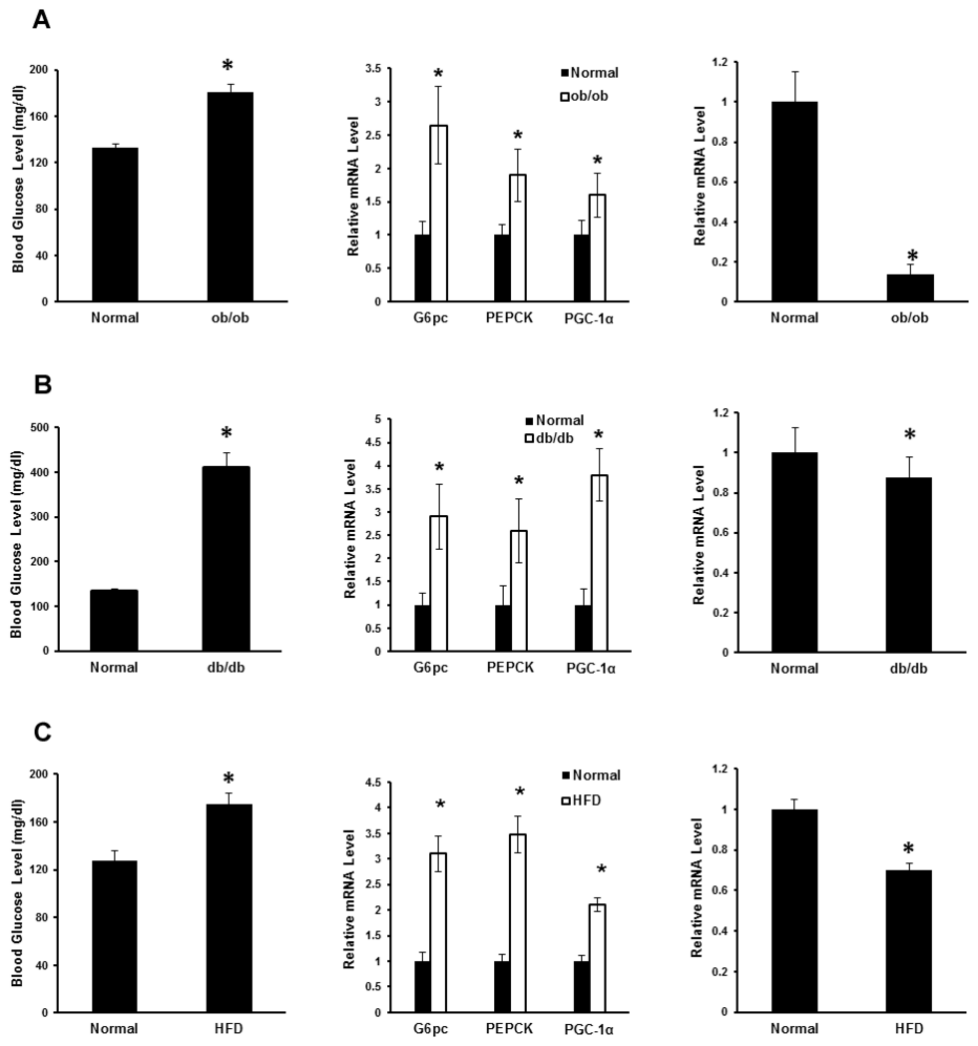
Expression levels of Tff3 in livers of mice with insulin resistance are decreased. (A) Blood glucose were measured (left panel) and mRNA levels of hepatic gluconeogenic genes (middle panel) and Tff3 gene (right panel) in C57BL/6J (control) and ob/ob mice (n=3) were measured by quantitative real-time PCR. (B) Blood glucose were measured (left panel) and mRNA levels of hepatic gluconeogenic genes (middle panel) and Tff3 gene (right panel) in C57BL/6J (control) and db/db mice (n=3) were measured by quantitative real-time PCR. (C) Normal C57BL/6J mice were fed normal chow (control) or fed a high-fat (HF) diet (DIO mice) for 16 weeks (n=3). Blood glucose levels were measured (left panel) and mRNA levels of hepatic gluconeogenic genes (middle panel) and Tff3 gene (right panel) were measured by quantitative real-time PCR. Data are mean ± SEM. *P < 0.05.

To identify novel factors involved in dysfunctional hepatic glucose homeostasis in obesity, we performed mRNA microarray analysis on livers of ob/ob mice, a widely used obese model. Our preliminary microarray data indicated that hepatic Tff3 expression levels were decreased in ob/ob mice compared to control mice. We further measured the expression levels of Tff3 and gluconeogenic genes by real-time PCR in the livers of different mouse models, including ob/ob, db/db and high-fat diet-induced obese (DIO) mice. We found that blood glucose levels in ob/ob mice were higher than those in normal mice. Correspondingly, the mRNA levels of gluconeogenic genes such as G6pc, PEPCK and PGC-1α, were all up-regulated in ob/ob mice. However, the hepatic Tff3 mRNA level in ob/ob mice was lower than that in normal mice. Similar results were obtained in DIO and db/db mice (Figure 1).

### Adenovirus-mediated overexpression of Tff3 represses cellular glucose production in primary mouse hepatocytes

**Figure 2:**
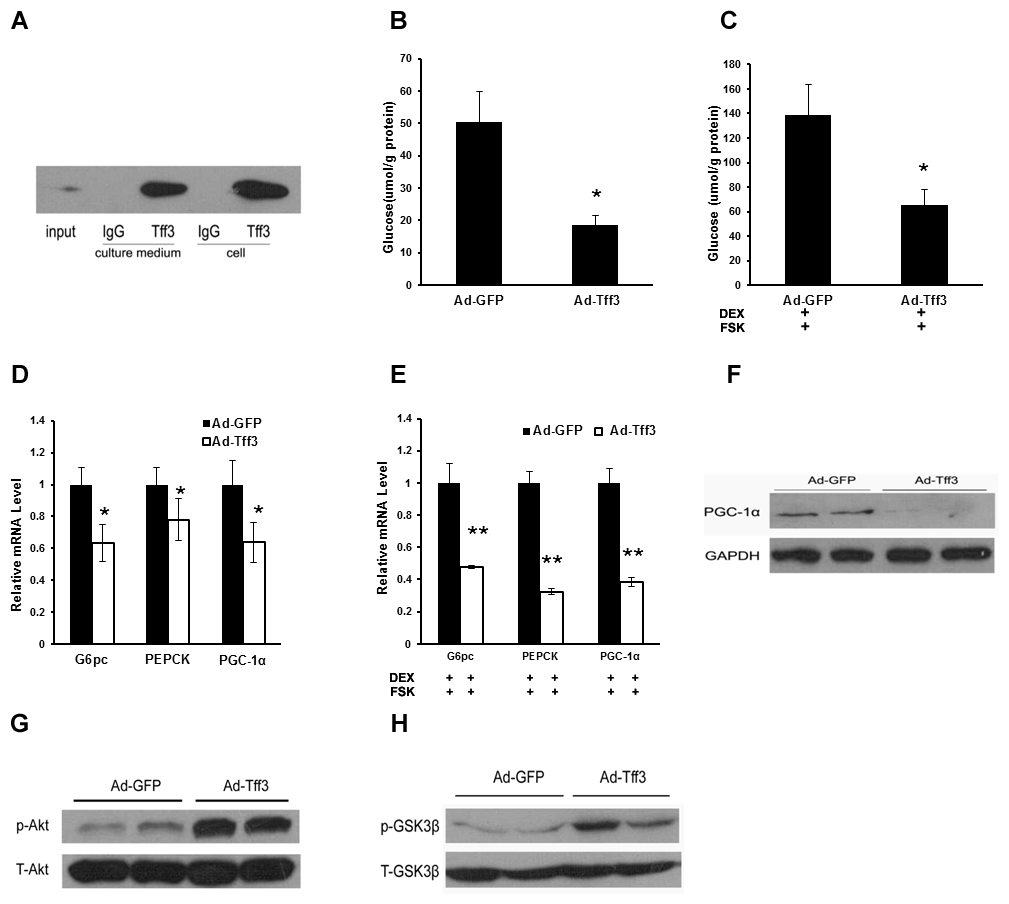
Adenovirus-mediated overexpression of Tff3 represses cellular glucose production in primary mouse hepatocytes. (A) Primary mouse hepatocytes were isolated and treated with adenovirus expressing GFP (Ad-GFP) or c-Myc-tagged Tff3 (Ad-Tff3) for 30h. Then cell extracts and culture medium were collected for immunoprecipitation with anti-Myc. Immunoprecipitates were immunoblotted with anti-Tff3. (B-C) Primary hepatocytes were treated with Ad-GFP or Ad-Tff3 for 30h. Then cells were washed with PBS and incubated in phenol red-free medium in the absence (B) or presence (C) of 1 µM DEX and 10 µM FSK for another 3h. Glucose concentrations in the medium were measured as described in Materials and Methods section. (D-E) Primary hepatocytes were treated with Ad-GFP or Ad-Tff3 for 30h. Then cells were incubated with or without 1 µM DEX and 10 µM FSK for another 1.5h. mRNA levels of gluconeogenic genes in cells were measured by quantitative real-time PCR analysis. (F) Primary mouse hepatocytes were treated as described in (A). Cell extracts were used for Western blotting analysis of PGC-1α protein level. (G-H) Mouse primary hepatocytes were treated with Ad-GFP or Ad-Tff3 for 24h, and then cells were incubated for at least 12h in serum-free medium. After that cells were treated with insulin for 15 min. Cells extracts were used for Western blotting analysis of insulin signaling molecules. Data are mean ±SEM. *P < 0.05, **P < 0.01.

To study the physiological functions of Tff3, we first generated adenovirus expressing c-Myc-tagged Tff3 (Ad-Tff3). Primary mouse hepatocytes were isolated and infected with Ad-Tff3. Culture medium and cell extracts were collected 30h after infection and were immunoprecipitated with anti-Myc. Then these immunoprecipitates were immunoblotted with anti-Tff3. As a result, Tff3 peptides were detected in both culture medium and cell extracts from Ad-Tff3-infected hepatocytes (Figure 2A), indicating Tff3 peptides were effectively secreted from primary hepatocytes. To study whether the Tff3 affected cellular glucose production, primary mouse hepatocytes were treated with Ad-GFP or Ad-Tff3 and glucose concentrations in the medium were measured. We found that overexpression of Tff3 significantly reduced cellular glucose output in the absence or presence of dexamethasone (DEX) and forskolin (FSK) (Figure 2B and 2C). Consistent with these results, the mRNA levels of gluconeogenic genes including PGC-1α，PEPCK and G6pc, were also decreased in Ad-Tff3-infected primary hepatocytes in the absence or presence of DEX and FSK (Figure 2D and 2E). Correspondingly, the protein levels of PGC-1α were also decreased, as revealed by immunoblotting assay (Figure 2F). Next, we studied whether alteration of Tff3 expression level in primary mouse hepatocytes would affect the insulin-signaling pathway. Indeed, we observed that overexpression of Tff3 enhanced phosphorylation of Akt and GSK3β, but without changes in total Akt and GSK3β level (Figure 2G and 2H).

### Overexpression of Tff3 in db/db mouse livers reduces the blood glucose level and improves glucose tolerance

**Figure 3:**
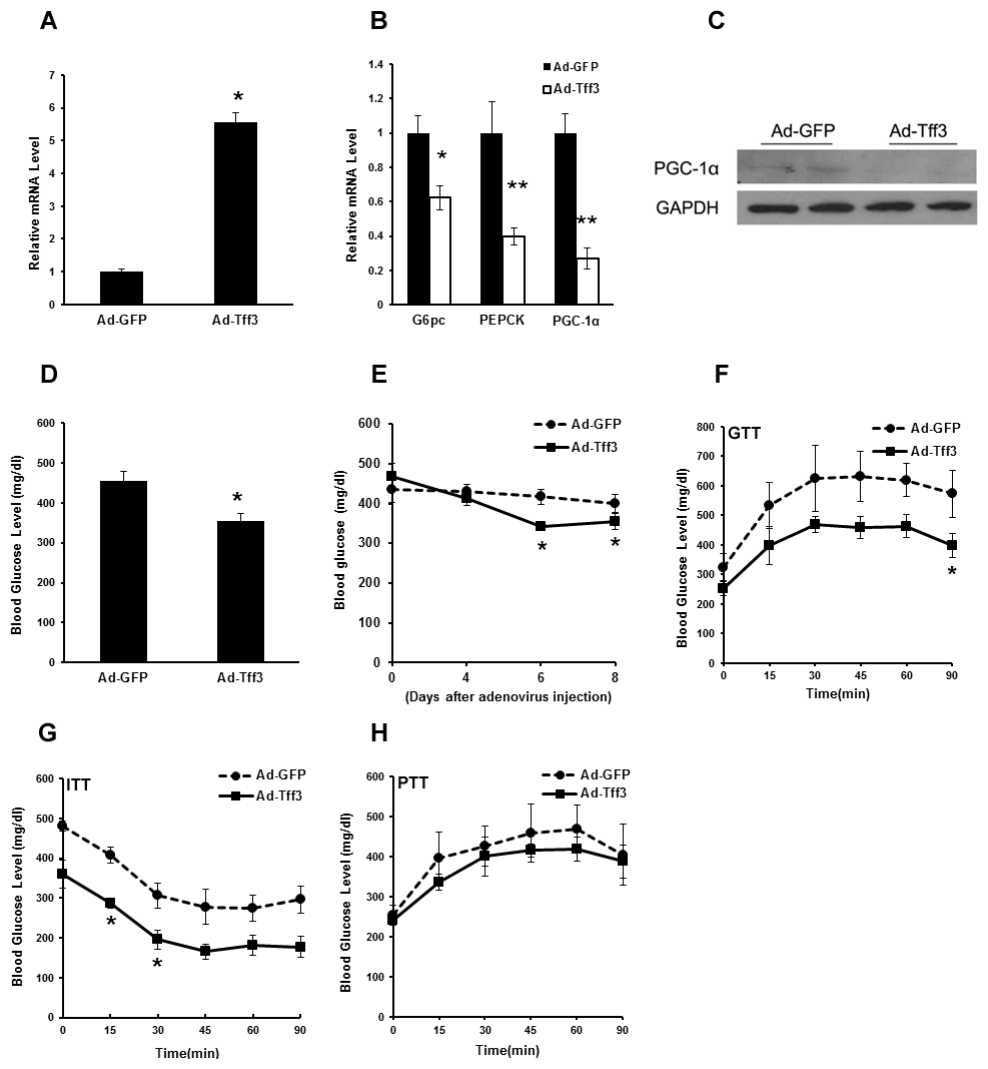
Overexpression of Tff3 reduces the blood glucose level and improves glucose tolerance in db/db mice. db/db mice were injected with Ad-GFP or Ad-Tff3. Mice were sacrificed for following analysis 5 days after infection. (A-B) Quantitative real-time PCR showing mRNA levels of Tff3 and gluconeogenic genes (G6pc, PEPCK and PGC-1α) in livers of db/db mice treated as above in the fasted states (n=5). (C) Western blot analysis of anti-PGC-1α protein levels in livers of db/db mice treated as described in (A-B). (D) Blood glucose levels in the same mice as in (A-B) (n=5). (E) Blood Glucose levels were measured in another groups of mice injected with Ad-GFP or Ad-Tff3 in different day (4, 6, 8 days) after infection. (F-H) Glucose tolerance tests (GTT) (F), insulin tolerance tests (ITT) (G) and pyruvate tolerance experiment (PTT) (H) in Ad-GFP or Ad-Tff3-injected db/db mice were performed 5 days after infection (n=4). Data are mean ±SEM. *P < 0.05, **P < 0.01.

Upon above results, it is reasonable to propose that Tff3 can inhibit the expression of hepatic gluconeogenic genes. To investigate this role of Tff3 in regulating hepatic glucose metabolism, Ad-Tff3 was purified and then injected into db/db mice via tail vein. 5 days after infection the mRNA level of Tff3 in liver of Ad-Tff3-infected mice were 5.4-fold higher than that in Ad-GFP-infected control mice (Figure 3A). The hepatic Tff3 overexpression decreased expression levels of G6PC, PEPCK and PGC-1α (Figure 3B and 3C). Consistent with these data, blood glucose levels in Ad-Tff3-infected mice were decreased (Figure 3D and 3E). To determine whether overexpression of Tff3 improves glucose intolerance and insulin sensitivity in these mice, glucose tolerance tests (GTT), Pyruvate tolerance test (PTT) and insulin tolerance tests (ITT) were performed. GTT experiments showed that blood glucose level at each time point tested in Ad-Tff3-infected mice were lower than that in Ad-GFP-infected control mice, suggesting that Tff3 activation improved glucose intolerance in db/db mice (Figure 3F). ITT experiments also suggested that Tff3 activation enhances insulin sensitivity (Figure 3G). However, PTT experiments indicated that glucose production in vivo was only modestly decreased by overexpression of Tff3 (Figure 3H).

### Overexpression of Tff3 in ob/ob mouse livers reduces the blood glucose level and improves glucose tolerance

**Figure 4:**
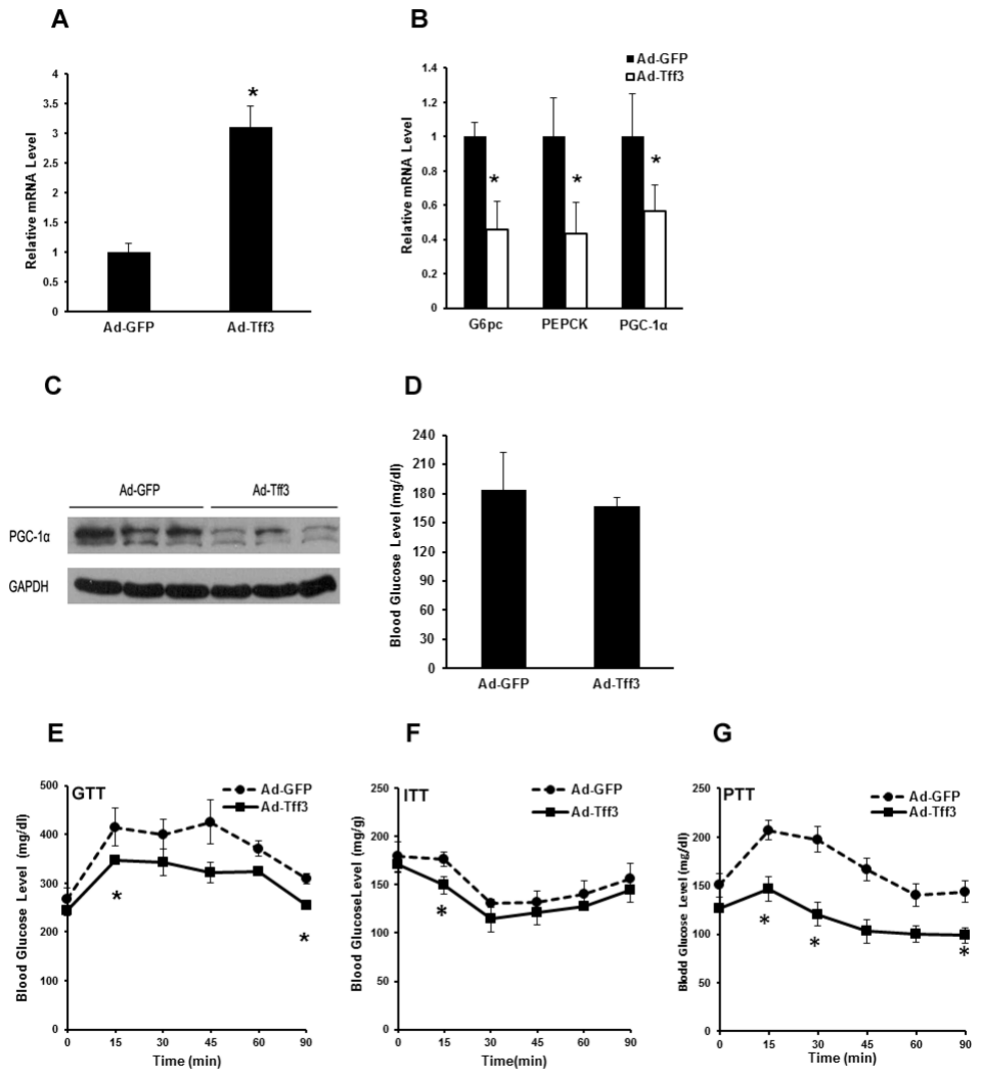
Overexpression of Tff3 reduces the blood glucose level and improves glucose tolerance in ob/ob mice. ob/ob mice were injected with Ad-GFP or Ad-Tff3. Mice were sacrificed for following analysis 5 days after infection. (A-B) Quantitative real-time PCR showing mRNA levels of Tff3 and gluconeogenic genes (G6pc, PEPCK and PGC-1α) in livers of ob/ob mice treated as above in the fasted states (n=5). (C) Western blot analysis of anti-PGC-1α protein levels in livers of ob/ob mice treated as described in (A-B). (D) Blood glucose levels in the same mice as in (A-B) (n=5). (E-G) Glucose tolerance tests (GTT) (E), insulin tolerance tests (ITT) (F) and pyruvate tolerance experiment (PTT) (G) in Ad-GFP or Ad-Tff3-injected ob/ob mice were performed 5 days after infection. Data are mean ±SEM. *P < 0.05.

Similar results were obtained on ob/ob mice. Injection of Ad-Tff3 into ob/ob mice led to an increase in Tff3 expression in ob/ob mouse livers (Figure 4A). Overexpression of Tff3 in ob/ob mice livers inhibited the expression of gluconeogenic genes (Figure 4B and 4C). However, overexpression of Tff3 in ob/ob mice just minimally but consistently decreased fasting glucose levels (Figure 4D). GTT experiments indicated that overexpression of Tff3 improved glucose intolerance (Figure 4E), while ITT and PTT experiments suggested that Tff3 activation modestly enhanced insulin sensitivity and decreased glucose production in vivo in ob/ob mice, respectively (Figure 4F and 4G).

### Overexpression of Tff3 improves insulin-resistance in DIO mice

**Figure 5:**
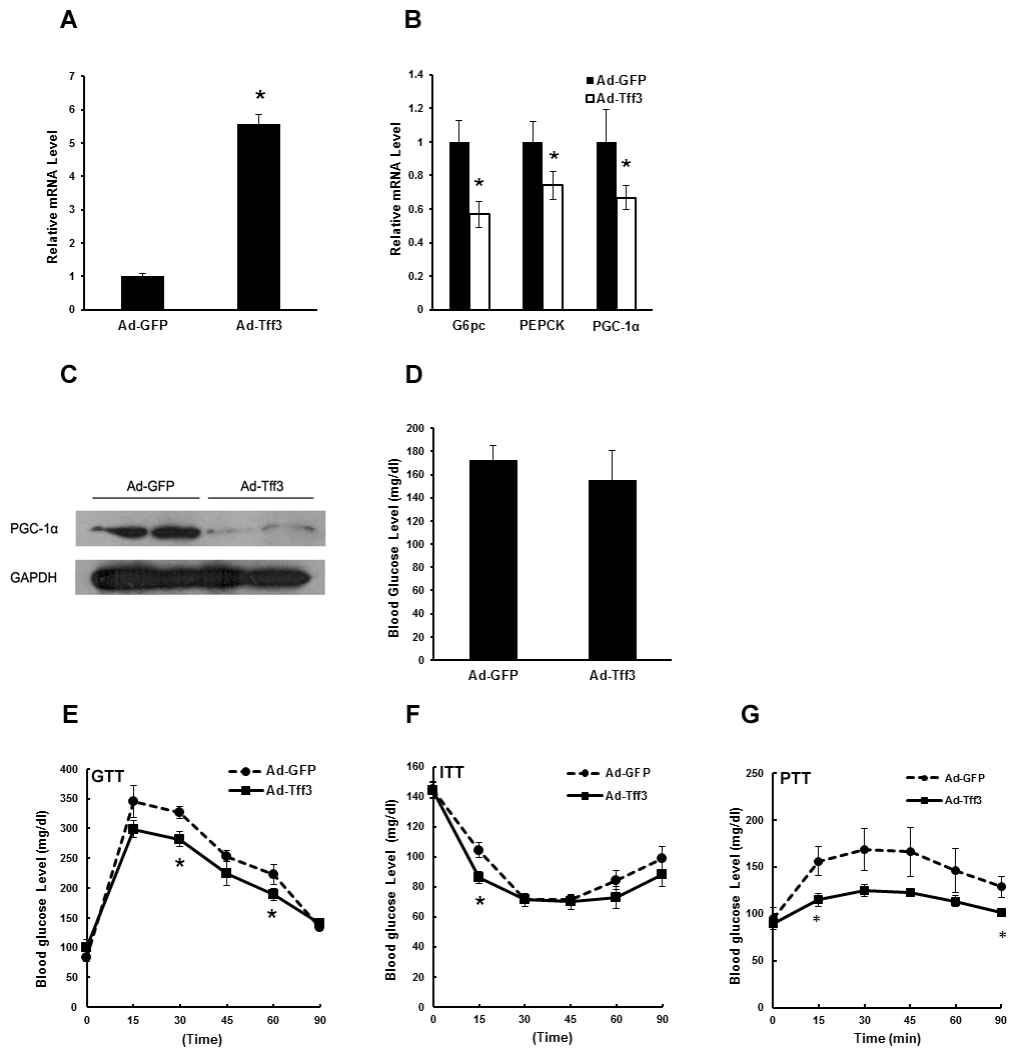
Overexpression of Tff3 improves glucose tolerance in DIO mice. Diet-induce-obesity (DIO) mice were injected with Ad-GFP or Ad-Tff3. Mice were sacrificed for following analysis 5 days after infection. (A-B) Quantitative real-time PCR showing mRNA levels of Tff3 and gluconeogenic genes (G6pc, PEPCK and PGC-1α) in livers of ob/ob mice treated as above in the fasted states (n=5). (C) Western blot analysis of anti-PGC-1α protein levels in livers of DIO mice treated as described in (A-B). (D) Blood glucose levels in the same mice as in (A-B) (n=5). (E-G) Glucose tolerance tests (GTT) (E), insulin tolerance tests (ITT) (F) and pyruvate tolerance experiment (PTT) (G) in Ad-GFP or Ad-Tff3-injected DIO mice were performed 5 days after infection (n=5). Data are mean ±SEM. *P < 0.05.

To eliminate concerns related to potential impact of leptin signaling deficiency in db/db or ob/ob mice, we examined the effects of Tff3 activation on glucose intolerance and insulin resistance in diet-induced obese (DIO) mice. Normal C57BL/6 mice (body weight: 20.25±0.32 gram; blood glucose levels: 96.9±4.88 mg/dl) were fed high-fat diet for more than 8 weeks and these mice displayed obesity and increased blood glucose levels (body weight: 37.94±1.80 gram; blood glucose levels: 169.2±4.23 mg/dl). Then these DIO mice were infected with Ad-GFP (control) or Ad-Tff3. Again, expression level of Tff3 and gluconeogenic genes were detected by real-time PCR (Figure 5A and 5B). Ad-Tff3-infected mice displayed increased hepatic Tff3 expression and Tff3 activation inhibited expression of gluconeogenic genes. PGC-1α protein levels in DIO mouse livers were also detected. As a result, we found that the protein levels of PGC-1α were decreased in livers of Ad-Tff3-infected mice compared to Ad-GFP-infected mice (Figure 5C). Blood glucose levels in Ad-Tff3-infected DIO mice were minimally decreased (Figure 5D). Ad-Tff3-infected DIO mice showed improvement in glucose tolerance (Figure 5E), as revealed by GTT experiments. Again, ITT and PTT experiments suggested that Tff3 activation modestly enhanced insulin sensitivity and decreased glucose production in vivo in DIO mice, respectively (Figure 5F and 5G).

## Discussion

Insulin resistance is a risk factor for the development of T2DM [26]. Hepatic insulin resistance causes abnormal activation of hepatic glucose production (HGP) and development of hyperglycemia. Thus, reducing hepatic gluconeogenesis and enhancing hepatic insulin sensitivity may meliorate hyperglycemia [8].

The liver is the site for producing various secretory proteins which play prominent roles in regulating glucose homeostasis and maintaining insulin sensitivity. For example, adropin as a secretory peptide from liver regulates the expression of hepatic lipogenic genes and improves glucose homeostasis [27]. In this study, we found that the expression levels of Tff3, a secretory protein which is the most widely expressed peptide in the TFF family in multiple tissues, were reduced in liver of mice with insulin resistance. Previous study demonstrates that nutritional status affects Tff3 expression levels in rat intestine: fasting decreases Tff3 mRNA level in duodenum and jejunum [24]. Another study also implies that hepatic Tff3 expression levels are associated with hepatic steatosis [28]. Additionally, glucose and insulin enhance Tff3 expression in intestinal epithelial cells (HT-29), whereas short-chain fatty acids inhibit Tff3 expression in mucous cells [29,30]. These studies suggest that Tff3 may be involved in glucose or lipids metabolism.

In this study we explored the function of Tff3 in regulating hepatic glucose metabolism. We firstly treated primary hepatocytes with adenovirus expressing Tff3 and found that overexpression of Tff3 decreased cellular glucose output and inhibited the expression of gluconeogenic genes such as G6pc, PEPCK and PGC-1α. Adenovirus-mediated overexpression of Tff3 in db/db mice decreased blood glucose levels and expression levels of hepatic gluconeogenic genes. GTT experiments indicated that Tff3 activation in these mice can improve glucose tolerance.

In mammals blood glucose levels are maintained within a very narrow range by the balance between glucose uptake by peripheral tissues and glucose secretion by the liver. In the fasted state, circulating glucagon levels increase to induce PGC-1α gene expression via CREB/TORC pathway [9,31,32]. In turn, induced PGC-1α stimulates expression of PEPCK and G6Pase genes, encoding key enzymes in the gluconeogenesis, through directly interacting to and coactivating transcriptional factor HNF4, FOXO1 and GR [9,33]. In contrast, after a meal, serum insulin levels increase, thereby inducing glucose uptake by peripheral tissues and blocking glucose production by liver. Insulin/Akt can suppress PGC-1α activity by several mechanisms. Insulin decreases PGC-1α expression in primary hepatocytes [32] and Akt can directly phosphorylate PGC-1α, inhibiting its activity [34]. Meanwhile, Akt can also phosphorylate FOXO1, inducing FOXO1 translocation to the cytoplasm and thereby decreasing its transcriptional activity [35].

Previous studies suggest that Tff3 can activate PI3K/Akt signaling pathway in different type of cells [15,16]. In this study we found that Tff3 activation also directly enhanced insulin signaling and stimulated phosphorylation of Akt in primary hepatocytes. Thus, it is reasonable to speculate that secreted Tff3 proteins from hepatocytes might decrease expression of PGC-1α and its target genes PEPCK and G6Pase and block hepatic glucose production through activating Akt signaling pathway.

The current evidences indicate that some immune cells, especially M1-like macrophages, in adipose tissue in obese humans and rodents accumulate to secrete a variety of cytokines, such as TNFα and interleuin-6, that cause decreased insulin sensitivity through both paracrine and endocrine mechanisms [36,37]. Genetic deletion of macrophage inflammatory pathway components protects against obesity-induced insulin resistance and glucose intolerance [38,39]. Based on the facts that adenovirus can also effectively infect immune cells, including macrophage [40,41], we cannot rule out the possibility that injected adenovirus expressing Tff3 infected immune cells and decreased cytokine secretions, thereby improving glucose intolerance in diabetic mice. Of note, although overexpression of Tff3 in primary hepatocytes significantly decreased expression of gluconeogenic genes and cellular glucose output, pyruvate tolerance tests (PTT) performed in db/db mice indicate that Ad-Tff3-infected mice displayed modestly reduced glucose production in vivo. Because kidney is also an important organ contributing to gluconeogenesis, we measured Tff3 expression levels in kidney of Ad-Tff3-infected mice. However, we did not observe the changes in renal Tff3 mRNA levels in these mice compared to Ad-GFP-infected control mice (data not shown). Thus, a possible explanation is that kidney gluconeogenesis is not impaired by Ad-Tff3, which produces glucose to compensate for the inhibitory effects of Tff3 on hepatic glucose production.

Additionally, although hepatic Tff3 mRNA levels are dramatically reduced in ob/ob mice, this reduction is modest in db/db mice. However, overexpression of Tff3 in db/db markedly decreased fasting blood glucose levels, while just minimal effects of Tff3 were observed in ob/ob or DIO mice. Additionally, overexpression of Tff3 in db/db mice significantly improved insulin sensitivity (as revealed by ITT experiments), whereas which is not observed in ob/ob and DIO mice. These differences might be caused by different genetic background of these mice.

The molecular mechanisms of Tff3 action are still unclear. Though no binding molecule has been identified with the classical characteristics of a receptor [12], we speculate that Tff3 may act through a receptor-mediated way. Additional studies are required to identify putative Tff3 receptor in the future.

In conclusion, we reveal that Tff3 activation improves glucose tolerance in diabetic mice. Tff3 alleviates hyperglycemia in diabetic mice by suppressing hepatic gluconeogenesis. Therefore, this study may provide a promising new lead for developing therapies against the metabolic disorders associated with T2DM.

## Supporting Information

Table S1 **Primer used in real-time PCR.**
(DOC)
