# Supplementary material for: Tff3, as a Novel Peptide, Regulates Hepatic Glucose Metabolism
Source: PLoS One. 2013 Sep 23;8(9):e75240. doi: 10.1371/journal.pone.0075240 (PMC3781022; doi:10.1371/journal.pone.0075240)
Supplement: Table S1 — Primer used in real-time PCR. (DOC) [file pone.0075240.s001.doc]

**Table S1**

Primer used in real-time PCR:

PGC-1α: Forward: TGGAGTGACATAGAGTGTGCTGC

Reverse: CTCAAATATGTTCGCAGGCTCA

PEPCK: Forward: CAGGATCGAAAGCAAGACAGT

Reverse: AAGTCCTCTTCCGACATCCAG

G6pc: Forward: GACTGGTTCAACCTCGTCTTC

Reverse: GTCTCACAGGTGACAGGGAAC

Tff3: Forward: GAAGCTTGCCTGCTGCCATG

Reverse: GCTTCAAAATGTGCATTCTG

β-actin: Forward: GTGCGACGAAGACGAGAC

Reverse: CCTGGTCGGTTTGATGTT
